# ARFID InitiativE Sweden (ARIES): study protocol for a large-scale genetic and registry-linked cohort study on avoidant/restrictive food intake disorder

**DOI:** 10.1136/bmjopen-2024-095559

**Published:** 2025-04-17

**Authors:** Liv Hog, Bengt T Fundin, Erik Everett Palm, Annelie Billger, Cynthia M Bulik, Afrouz Abbaspour, Lisa Dinkler

**Affiliations:** 1 Department of Medical Epidemiology and Biostatistics, Karolinska Institute, Stockholm, Sweden; 2 Department of Psychiatry, The University of North Carolina, Chapel Hill, North Carolina, USA; 3 Department of Nutrition, The University of North Carolina, Chapel Hill, North Carolina, USA

**Keywords:** GENETICS, PSYCHIATRY, MENTAL HEALTH, Eating disorders

## Abstract

**Abstract:**

**Introduction:**

The ARFID InitiativE Sweden (ARIES) investigates the genetic and environmental factors contributing to avoidant/restrictive food intake disorder (ARFID) in children and adolescents aged 6–14 years. ARIES will establish a national biobank and research registry. It aims to provide data for immediate research and track ARFID outcomes and clarify genetic links between ARFID and other conditions and analyse the gut microbiome to guide nutrition interventions.

**Methods and analysis:**

The study will involve 1500 Swedish children and adolescents with ARFID and a control group of 500 Swedish children and adolescents without ARFID. Parents/guardians and their children will complete online questionnaires assessing ARFID and other eating disorder (ED) pathology, co-occurring conditions, quality of life and parental stress and ED pathology. All participants will provide a saliva sample for comprehensive genetic analyses. Additionally, a subset of participants will provide a stool sample to investigate the gut microbiome in ARFID.

**Ethics and dissemination:**

ARIES was approved by the Swedish Ethical Review Authority (Dnr 2023-04638). All participants will give assent and their parents will complete informed consent. Data will be made available by the authors on reasonable request. Findings will be published in scientific journals and shared with the public and stakeholders in accessible ways, for example, via social media.

**STRENGTHS AND LIMITATIONS OF THIS STUDY**

- The ARFID InitiativE Sweden (ARIES) will be the largest epidemiological data collection of phenotype and genotype data in children and adolescents (6–14 years) with and without avoidant/restrictive food intake disorder (ARFID) to date.
- ARIES will establish a national research registry and biobank of childhood ARFID cases and controls containing comprehensive phenotype and genotype data enabling a wealth of research studies for many years to come.
- ARIES will be linked to Swedish population health and quality registers and, therefore, enable longitudinal follow-up to plot the course of ARFID and identify predictors of long-term outcome.
- The assessment of the ARFID phenotype relies on parent-reported questionnaires rather than clinical diagnoses, which allows for broader data collection in community settings where clinical diagnoses and structured diagnostic interviews may not always be available or feasible.
- Although the genome-wide association study (GWAS) sample size will be relatively small, integrating ARIES data with other ongoing collections globally will maximise statistical power for a coordinated GWAS meta-analysis, ensuring the most comprehensive and robust analysis to date and resulting in the largest ARFID GWAS conducted so far.

## Background

 The ARFID InitiativE Sweden (ARIES) study is designed to investigate genetic and environmental factors influencing avoidant/restrictive food intake disorder (ARFID) in children and adolescents between ages 6 and 14 years. ARIES is based on previous and ongoing research by the Eating Disorders Working Group of the Psychiatric Genomics Consortium (PGC-ED) including genome-wide association studies (GWASs) focused on the genetic basis of ARFID,^1^ anorexia nervosa (AN),^2^ bulimia nervosa (BN) and binge-eating disorder (BED).^3 4^ The overall aim is to explicate the genetic architecture of all EDs; their relationship to each other and to other psychiatric, neurodevelopmental, and somatic conditions and traits; and, ultimately, to facilitate a genetically informed nosology of EDs.

ARIES will contribute to this effort by providing detailed phenotype and genotype information of a large sample of children and adolescents with and without ARFID in Sweden. Using a subset of the sample, ARIES will provide information on the gut microbial environment in ARFID. ARIES will clarify within-disorder heterogeneity; identify genetic, environmental and microbial risk factors and correlates of the condition, and investigate the relation of ARFID to other EDs and psychiatric, neurodevelopmental and somatic conditions. The ultimate goal of this work is to provide important aetiological information to inform the development of effective interventions for ARFID.

ARFID is present in 1%–2% of the general population^5 6^ and was formally recognised in 2013.^7^ ARFID is a feeding and eating disorder characterised by an extremely restricted dietary quantity or variety resulting in significant weight loss, nutritional deficiencies, reliance on nutritional supplements and/or psychosocial impairment.^7^ In contrast to other EDs, ARFID occurs in approximately equal proportions of males and females,^6 8^ has an earlier age of onset^9^ and the restrictive eating is not primarily motivated by weight and shape concerns.^7^ ARFID is often accompanied by severe and sometimes life-threatening somatic and psychosocial comorbidities, leading to high social and financial burden for patients and their families.^10^,^14^ Despite the significant impairment, our knowledge on ARFID aetiology is limited. In part due to this insufficient understanding, evidence-based treatments are only starting to be developed^15^ and no specific treatment guidelines exist.^16^ A longer duration of untreated EDs other than ARFID has been found to decrease the likelihood of recovery.^17^ It is, therefore, likely that the lack of treatment options significantly contributes to the chronicity of ARFID.

ARFID is conceptualised as a single disorder; however, the Diagnostic and Statistical Manual of Mental Disorders fifth edition (DSM-5) describes three non-mutually exclusive presentations: (1) sensory-based food avoidance; (2) low appetite or interest in food and (3) fear of aversive consequences of eating. These symptom clusters overlap in a subset of individuals with ARFID (15%–51%).^818^,^20^ Comorbidities vary somewhat depending on the dominant presentation(s)^11 19^; for instance, sensory-based food avoidance has been shown to be more strongly related to neurodevelopmental conditions, low appetite or interest in food to mood disorders,^21^ and fear of aversive consequences to obsessive-compulsive disorder (OCD) and anxiety disorders.^11^

ARFID-related phenotypes such as appetite, food fussiness and food neophobia have been shown to be moderately to highly heritable.^22^,^25^ Our previous research using Swedish twin data further supports our aim of conducting a GWAS for ARFID: with a twin-based heritability of 70%–85%,^5^ ARFID is among the most heritable of EDs^26^ and of psychiatric conditions in general.^27^,^30^

In addition to identifying genetic risk factors for ARFID, ARIES is also exploring the role of the gut microbes (microbiota), along with their genes, collectively known as the gut microbiome. Disruption of the gut microbiota (dysbiosis) has been found both in children with severe malnutrition^31^ and in patients with AN^32^,^35^ and is commonly associated with cardiovascular diseases,^36^ cancer,^37^ neurological diseases^38^ and psychiatric conditions, such as anxiety and depression.^39^ Moreover, malnutrition in childhood has been found to be causally associated with immature gut microbiota.^31^ Nutrient intake and dietary variety during early childhood are essential for the establishment of rich and healthy gut microbiota,^40 41^ with the introduction of solid food being an important step towards the emergence of a complex and more adult-like gut microbiota.^40 42^ Prolonged restriction of food quantity and/or variety is likely to negatively impact the composition and function of the gut microbiota in ARFID. We, therefore, intend to study the association between ARFID-related eating habits and alterations in the gut microbiome.

### Aims

The overall aim of ARIES is to identify genetic, environmental and gut microbial factors associated with ARFID, with the goal of developing a comprehensive aetiological model. We will also characterise the phenotypic and genetic associations of ARFID with other EDs, as well as with psychiatric, neurodevelopmental, and somatic conditions and other relevant phenotypes (eg, metabolic, anthropometric). In this context, we will create a data repository comprising (1) parent-reported and self-reported phenotypic information on eating behaviours, co-occurring conditions, environmental exposures, health-related quality of life and healthcare utilisation and (2) a biorepository including saliva samples for genomic studies and stool samples for study of the gut microbiota. Both will be linked with Swedish health registers^43^,^45^ and the National Quality Register for Specialized Eating Disorder Treatment.^46^

#### Aim 1: characterise phenotypic features of ARFID

We will collect phenotypic information via online questionnaires and connect it with population health and quality registers to analyse demographics; symptom patterns; onset; disorder course; food intake; co-occurring psychiatric, neurodevelopmental and somatic conditions; environmental exposures; health-related quality of life and healthcare utilisation in people with ARFID. For most of these variables, we will also conduct analyses to compare ARFID cases with controls.

##### Hypotheses

Most participants with ARFID will show early childhood onset and a persistent course of illness. Furthermore, we expect participants with ARFID to report more co-occurring psychiatric, neurodevelopmental and somatic conditions than participants without ARFID. In terms of ARFID presentations, we expect to find sensory-based food avoidance to be related to autism, low appetite to depression and metabolic factors, and fear-based avoidance to anxiety and OCD.

### Aim 2: conduct a within-disorder ARFID GWAS

We will extract DNA from saliva samples provided by all participating children and adolescents. We will then (1) conduct a GWAS of the ARIES sample, (2) conduct a GWAS meta-analysis including other ARFID samples collected by the PGC-ED and (3) conduct a standard suite of genomic analyses.

#### Hypotheses

GWAS is a hypothesis-free method to identify genetic loci across the entire genome that are statistically associated with risk for a trait or a condition. We expect to identify genome-wide significant loci for ARFID.

### Aim 3: define the genetic relation of ARFID with other EDs

We will conduct a suite of analyses comparing ARFID to other EDs (AN, BN and BED) to examine their interrelations and investigate the overall genetic architecture of ARFID in comparison to other EDs.

#### Hypotheses

ARFID will share a core set of genetic factors with other EDs and be differentiated by disorder-specific genetic factors. ARFID will show the strongest genetic overlap with AN.

### Aim 4: define the genetic relation of ARFID with psychiatric, neurodevelopmental, somatic and other relevant phenotypes

We will conduct a suite of analyses comparing ARFID to psychiatric, neurodevelopmental, somatic and other relevant phenotypes to examine their interrelations and investigate the overall genetic architecture of ARFID in comparison to these phenotypes.

#### Hypotheses

Sensory-based food avoidance will be genetically related to autism, low appetite to depression and metabolic factors, and fear-based avoidance to anxiety and OCD. We also expect the genetic associations of ARFID with neurodevelopmental conditions and anxiety to be stronger than the genetic associations of ARFID with other EDs.

### Aim 5: explore the composition, diversity, maturity and function of the gut microbiome in participants with ARFID

We will collect and sequence stool samples from 100 cases and 100 controls to characterise the gut microbiomes and identify microbial genes and pathways associated with ARFID-related dietary, psychiatric, clinical and other relevant phenotypic factors.

#### Hypotheses

ARFID will be associated with reduced overall microbial diversity (similar to other EDs like AN), and immaturity of the gut microbiota. Specific microbial taxa, genes and pathways will be linked to ARFID pathology, dietary patterns and other ARFID-related phenotypes in participants with ARFID. For example, given that ARFID can involve selective eating habits, we expect to find changes in the levels of bacterial genera associated with diets characterised by specific macronutrient composition such as *Bacteroides* (associated with a diet high in animal proteins and fats) or *Prevotella* (associated with a diet high in carbohydrates and fibre) depending on specific dietary restrictions of individuals with ARFID.

## Methods

### Parent Advisory Council

A Parent Advisory Council (PAC) comprising eight parents/guardians of children and adolescents with ARFID will accompany our study to ensure maximum benefit on the community and support of parents/guardians of children with ARFID. Prior to the main study launch, the PAC completed a beta version of the entire study, including all questionnaires and saliva sampling and provided feedback on all study procedures.

### Participants

#### Objective

We will recruit 1500 children and adolescents aged 6–14 years with ARFID and 500 age-matched controls from Sweden to be included in the main study. 100 children and adolescents aged 6–14 years with ARFID, and 100 age-matched controls will also be selected and included in the microbiome substudy.

#### Recruitment

Study recruitment began in May 2024, and we anticipate closing recruitment in summer 2025. To ascertain cases, we developed and launched a campaign for information dissemination and recruitment using a variety of different methods, including social media, healthcare centres (eg, ED clinics, child and adolescent psychiatry, adult psychiatry and paediatric services), school healthcare and advocacy organisations for EDs and neurodevelopmental conditions. The Centre for Eating Disorders Innovation (CEDI) Social Media Committee assists in designing a campaign of informational material (print, audio and video) containing education about ARFID and invitations to participate in research. To ensure recruitment of a group of participants who represent ARFID distribution in the population, we include drawings of children and adolescents of different genders, ages, bodies and ancestries. To further increase reach, we recruit community members and influencers to promote ARIES. We focus the first part of our recruitment campaign to ascertain ARFID cases and the second part of our recruitment campaign to ascertain ARFID controls.

### Procedure

Parents/guardians of children and adolescents with ARFID who are interested in participation will be referred to the study website (www.ariesstudy.se) that provides detailed study information and contact details in case of questions (‘parents/guardians’ will henceforth be referred to as ‘parents’ for readability). To be eligible for participation, all individuals must have a valid Swedish personal number (a unique national identification number), which is required for both providing consent and linking to the Swedish National Health Registers. Study information is provided in three versions: for children (6–11 years), for adolescents (12–14 years), and for parents. If interested in participation, parents can register their child and provide online informed consent on the study’s website. Parents will complete online screening questionnaires covering ARFID and other disordered eating cognitions and behaviours including the Pica, ARFID and Rumination Disorder Interview-ARFID Questionnaire (PARDI-AR-Q),^47^ the Nine Item ARFID Screen (NIAS)^48^ and the parent version of the Eating Disorder Examination Questionnaire (PEDE-Q).^49^

The online screening questionnaires will be used to create a diagnostic algorithm for DSM-5 ARFID and determine eligibility and case status of the child. If the child is eligible for the study by parent-report, parents are instructed to complete additional questionnaires (some are mandatory, some are optional; online supplemental table S1). Additionally, we implemented optional self-report questionnaires for adolescents. A saliva sample collection kit including return packaging is mailed to the parent’s address with directions for saliva collection from the child. Those participating in the microbiome substudy will also receive a stool collection kit. Participation in the main study will be complete as soon as the kits are received by Karolinska Institutet (KI) Biobank and all mandatory questionnaires are completed. All participants receive two cinema tickets as soon as participation in the main study is complete. We do not provide participants in the microbiome substudy with additional reimbursement.

### Assessment battery

Online supplemental table S1 presents our assessment battery including validated instruments measuring demographics; ARFID symptoms; other ED pathology; food intake; co-occurring psychiatric, neurodevelopmental, and somatic conditions and traits; treatment utilization; environmental exposures; impairment and quality of life; and parental stress and ED pathology. All mandatory assessments are parent-reported; additionally, we offer optional questionnaires to be completed by parents and adolescents. Completion of the entire assessment battery will take approximately 90 minutes and can be done across several sittings. Questionnaires are applied in Swedish and have been adapted for administration via Forsta (previously Confirmit).

### Case definition

Cases will be participants who screen positive for ARFID on the PARDI-AR-Q or NIAS, report no clinically significant disordered eating behaviours in the last 28 days that could suggest the presence of AN or BN on PEDE-Q and have no lifetime diagnosis of AN or BN. Controls will be participants who screen negative for ARFID on PARDI-AR-Q and NIAS, report no clinically significant disordered eating in the last 28 days suggesting the presence of AN or BN on PEDE-Q, have no lifetime diagnosis of any ED or other psychiatric condition, and who have no first-degree relatives with an ED as reported by the parent. Case participants who will be included in the microbiome substudy will display exceptionally high scores on the PARDI-AR-Q subscale for sensory-based food avoidance (see table 1), and control participants will display exceptionally low scores on all PARDI-AR-Q and NIAS subscales. Table 1 presents detailed inclusion and exclusion criteria for cases and controls included in the main study and in the microbiome substudy.

**Table 1:** Inclusion and exclusion criteria for the main study and the microbiome substudy

	Main study		Microbiome substudy	
	Cases	Controls	Cases	Controls
NIAS	PE>9 **OR** LA>8 **OR** F>9	PE≤9 **AND** LA≤8 **AND** F≤9		PE<5 **AND** LA≤1 **AND** F≤1
	**OR**	**AND**		**AND**
PARDI-AR-Q	A0 met **AND** (A1 **OR** A2 **OR** A3 **OR** A4) met	A0—A4 not met	S>5.5*	S≤1 **AND** LA≤1 **AND** F≤1
	**AND**	**AND**	**AND**	**AND**
EDE-Q	Global score <4 **AND** No vomiting **AND** No laxative use	Global score <4 **AND** No vomiting **AND** No laxative use **AND** <5 binge eating episodes		
	**AND**	**AND**		
Control questions	No lifetime diagnosis of AN or BN	No lifetime diagnosis of any ED **AND** No first degree relative with a lifetime diagnosis of any ED **AND** No current diagnosis of a psychiatric disorder	No antibiotics or probiotics during the last 3 months **AND** Never hormones for gender reassignment **AND** No current inflammatory GI condition	No antibiotics or probiotics during the last 3 months **AND** Never hormones for gender reassignment **AND** No current inflammatory GI condition

* Due to the dominance of sensory-based food avoidance in children with ARFID^69^ and in order to obtain a sufficient sample size for microbiome analyses, we focused our microbiome analysis on those with high scores on S to investigate the association of sensory-based food avoidance with the gut microbiome.

AN, anorexia nervosa; ARFID, avoidant/restrictive food intake disorder; BN, bulimia nervosa; ED, eating disorder; EDE-Q, Eating Disorder Examination Questionnaire; F, fear score; GI, gastrointestinal; LA, low appetite score; NIAS, Nine Item ARFID Screen; PARDI-AR-Q, Pica, ARFID, and Rumination Disorder Interview-ARFID Questionnaire; PE, picky eating score; S, sensory-based food avoidance score.

### Biological samples

#### Saliva samples

Saliva sampling will be conducted using Oragene-DNA saliva self-collection kit (OG-500, DNA Genotek) by the participants at home and mailed back to KI Biobank. Parents will oversee the saliva collection. To guarantee safe transport and storage of the samples, the kits stabilise DNA at ambient temperature for several years.

#### Stool samples

Stool samples will be collected at home using the OMNIgene-GUT sample collection kit (OM-200, DNA Genotek) from participants included in the microbiome substudy. Parents will assist in conducting the stool collection and complete the Bristol Stool Scale.^50^ To guarantee safe transport and storage of the samples, the kits stabilise DNA at ambient temperature for 60 days. Prior to stool sampling, participants document their dietary intake in an online 24 hours food recall logbook.^51 52^

#### DNA extraction and genotyping

Saliva samples will be stored at KI Biobank. We will use standard DNA extraction protocols and GWAS genotyping via the Illumina global screening array chip, or optimal methods available at the time of genotyping. Cases and controls will be randomised to plates.

#### Microbial DNA extraction and metagenomic shotgun sequencing

Stool samples will be stored at KI Biobank. Microbial DNA extraction will be performed using a combination of physical disruption of the bacterial cells and chemical DNA purification. Whole genome shotgun sequencing will be performed using the Illumina NovaSeq platform to obtain high-resolution taxonomic and functional microbiome data. Raw sequence data will be quality filtered and trimmed to remove host DNA and bases with PHRED quality score <20, yielding analysis-ready data. As sequencing technology and bioinformatic tools continue to advance, we will use the most effective methods and tools available at the time of analysis.

### Additional data sources

Our data will be linked to nationwide Swedish health registers including the National Patient Register (NPR),^43^ the Prescribed Drug Register (PDR),^45^ the Medical Birth Register^44^ and the National Quality Register for Specialised Eating Disorder Treatment (Riksät/Stepwise).^46^ Register linkage is conducted by government agencies via the Swedish personal number. The NPR provides data on all inpatient care since 1987 and around 80% of specialised outpatient care since 2001,^43^ using International Classification of Diseases 10th revision classification since 1997. We will use the NPR to identify ARFID-related diagnoses; other EDs and psychiatric, neurodevelopmental and somatic conditions; as well as to track healthcare utilisation over time. Evidence shows strong agreement between ED diagnoses in the NPR and in Riksät/Stepwise, confirming the validity of NPR data for research.^53^ The PDR provides data on all drug prescriptions dispensed since 2005, with active drug ingredients categorised using the Anatomical Therapeutic Chemical (ATC) Classification System.^45^ We will use ATC codes to identify prescribed nutritional supplements and enteral nutrition relevant to ARFID and medication utilisation over time. Riksät/Stepwise contains data on ED treatment from around 100 specialised and general psychiatry units across 21 regions since 1999. We will extract diagnoses and clinical characteristics, such as symptom severity and impairment. Riksät offers broader coverage and includes DSM-IV subtypes, providing more extensive information than the NPR.^46^

For GWAS, we will combine our data with other completed, ongoing and planned collections conducted by PGC-ED. Refer to table 2 for the expected sample sizes at the time of analysis.

**Table 2:** Sample sizes and availability of genotypes for the ARFID-GWAS meta-analysis

Study	Cases	Controls	Age	Available by
ASTRI	300	⁓18 000	6–12	Available
ARIES	1500	500	6–14	May 2025
ARFID-GEN	⁓3000	/	6–99	September 2024
EDGI2	⁓3000	⁓1300	15–99	March 2026
PGC-ED	1200	⁓42 000	15–99	Available

ARFID, avoidant/restrictive food intake disorder; ARFID-GEN, ARFID–Genes and Environment; ARIES, ARFID InitiativE Sweden; ASTRI, ARFID in the Swedish Twin Registry Initiative; EDGI2, Eating Disorders Genetics Initiative 2; GWAS, genome-wide association studies; PGC-ED, Eating Disorders Working Group of the Psychiatric Genomics Consortium.

### Planned data analysis

#### Data management

Data will be stored in accordance with KI rules for data protection. Identifying personal information will be stored with very high security and strict physical and computer access control. ARIES data will be part of the master database CEDIX (all data at CEDI). Participants are not identifiable other than by the research group. Prior to analysis, data are pseudonymised. The key connecting ID to participant identity is only available at the Department of Medical Epidemiology and Biostatistics at KI. To protect privacy, we will practise cell suppression and not report data on subgroups with fewer than five individuals.

#### Statistical power

For the phenotypic analyses, we would need 394 participants in both case and control group to detect a small effect size (0.2) at an alpha level of 0.5 and 80% power and are, therefore, well powered.

For GWAS analyses, we set α=5×10^−8^. GWAS meta-analysis will include >71 000 samples (9000 cases; 62 360 controls; table 2) and will have >80% power to detect common variants (minor allele frequency [MAF]>5%) with modest effect sizes (OR>1.25), and low frequency variants (MAF>1%) with moderate effect sizes (OR>1.59). We anticipate detecting medium to large genetic effects for ARFID, considering a twin-based heritability estimate of 79%^5^ and are, therefore, adequately powered. No published studies exist on the gut microbiota in ARFID to guide our power analyses. However, our given sample size of 100 cases and 100 controls exceeds sample sizes of most published studies on the gut microbiota in AN. We expect to be adequately powered to detect medium to large effect sizes.

#### Data analysis

Research data will be pseudonymised for data analyses. We will follow predetermined statistical analysis plans for phenotype, genotype and gut microbiome data. All analyses will include age and sex/gender as covariates and be corrected for multiple testing.

##### Phenotypic analyses

We will describe the ARFID sample to present demographics; ARFID symptoms, onset and course; other ED pathology; food intake; co-occurring psychiatric, neurodevelopmental and somatic conditions and traits; treatment utilisation; environmental exposures; impairment and quality of life; and parental stress and ED pathology. Depending on the distribution, we will compute mean/SD or median/IQR for continuous variables, and *N*/percent for categorical variables. To compare ARFID cases with controls on all outcomes, we will conduct generalised linear modelling.

##### Genetic analysis

We will use the Ricopili software pipeline (Rapid Imputation for Consortias Pipeline, developed by PGC) including modules for preimputation (QC), principal component analysis, imputation and meta-analysis to process hundreds of GWAS data sets rapidly and consistently.^54^

First, we will conduct an independent within-disorder GWAS of the ARIES sample alone and then combine the ARIES sample with additional ARFID samples from the PGC-ED to conduct a GWAS meta-analysis. Post-GWAS analyses will include, among others, the analysis of X chromosome (chrX) variants; secondary GWAS analyses separately on females and males to evaluate the similarity to the results of the primary GWAS; clumping GWAS to convert significant single nucleotide polymorphisms (SNPs) (p<5×10^−8^) into regions; conditional and joint analyses; functional genomic integration; linkage disequilibrium score regression (LDSC) to estimate SNP-based heritabilities^55 56^; the calculation of rare copy number variants (CNVs) and CNV burden; the calculation of polygenic risk scores (PRS) using the p-thresholding method and a summary statistics version of Bayesian multiple regression (SBayesR)^57^; an ARFID presentation analysis examining the association between ARFID PRS and PARDI-AR-Q subscale scores sensory-based food avoidance, lack of interest in food or eating and fear; gene-wise tests of association with ARFID using multimarker analysis of genomic annotation^58^; the investigation of partitioned heritability using stratified LDSC,^59^ and whether genes associated with ARFID are enriched in certain tissues, cell types and pathways.

We will compare disorder-specific GWAS for ARFID, AN, BN and BED and transdiagnostic behaviours (eg, restriction, binge eating). Analyses will include, among others, the calculation of common variant-based genetic correlations (SNP-r_g_s) for ARFID and selected traits to investigate common genetic variation using GWAS summary statistics; generalised summary data-based Mendelian randomisation to investigate causal relationships between ARFID and selected traits; multitrait-based conditional and joint analysis (GCTA-mtCOJO)^60^ to investigate whether SNP associations for EDs can be traced back to their relationship with correlated traits such as type 2 diabetes, body mass index and depression; the calculation of disorder-specific SNP associations by conducting a case vs case GWAS (eg, ARFID vs BED) and multipolygenic scores^61^ by using the largest available GWAS summary statistics of psychiatric and somatic conditions and traits to predict selected outcomes (ARFID, AN, BN, BED); and genomic structural equation modelling^62^ to investigate the genetic architecture of ARFID compared with other EDs.

Finally, we will analyse whether ARFID is genetically associated with various psychiatric, neurodevelopmental, somatic and other relevant phenotypes by making use of the same methods outlined above. Summary statistics of selected traits are provided by other PGC working groups and the UK Biobank.

##### Gut microbiome analysis

Taxonomic profile will be characterised using Kraken2,^63^ and metabolic profile will be characterised through the HUMAnN2 pipeline.^64^ We will calculate α-diversity (within-sample diversity) using the Shannon Index to account for both richness and evenness within the dataset. A sensitivity analysis will be conducted to test the effects of using richness and Pielou’s evenness. For β-diversity (between-sample diversity), we will generate distance matrices using both phylogenetic (weighted and unweighted UniFrac) and non-phylogenetic (Bray-Curtis, Jaccard, Aitchison) indices. These matrices will be used to quantify differences in gut microbiome profiles between cases and controls via permutational multivariate analysis of variance and permutational multivariate analysis of dispersion.^65 66^ For the differential abundance analysis, we will describe differences in the mean abundance of microbial taxa and genes by using analysis of composition of microbiomes.^67^ Associations between phenotypic measures—including dietary, psychological, metabolic and clinical measures—and microbial and dietary diversity will be reported using linear models. Using a random forest machine learning model,^31^ we will regress the relative abundance of microbial taxa against the chronological age of each child at the time of stool sample collection. The predicted microbiota ages of the participating children and adolescents will then be compared with the median microbiota age of chronologically age-matched children and adolescents in the control group to generate microbiota-for-age Z-scores.

## Discussion

In addition to an in-depth study of genetic and environmental factors associated with ARFID, ARIES will form the basis of a national research registry and biobank of childhood ARFID cases and controls. The cohort will provide immediate data and samples to address our baseline scientific aims and enable longitudinal follow-up to plot the course of ARFID and identify predictors of long-term outcome. The ARIES repository will be an invaluable resource for investigators who apply for access to questionnaire data, biological samples and participants who will have agreed to re-contact for research. With linkage to population health and quality registers, ARIES will be a unique resource and will be well-positioned to address essential questions about this pernicious and understudied illness.

We will also use our combined ARIES/PGC-ED GWAS data in cross-disorder analyses to determine how ARFID is best positioned genetically relative to other EDs, other psychiatric conditions, such as depression, anxiety and OCD, and to neurodevelopmental conditions, such as autism and attention deficit/hyperactivity disorder. Our unique inclusion of microbial data will reveal much about the impact of ARFID on the gut microbiota and microbiome and may inform the development of nutrition interventions for ARFID (eg, nutrigenomics, microbiota-directed complementary foods).

Furthermore, we will be able to use the genotyping array data to generate CNV calls. Since rare genetic CNVs play a role in neurodevelopmental conditions,^68^ we may find the same in ARFID. Our ARFID Biobank will also be poised for future exome or genome sequencing. The proposed work provides a rich foundation for decades of study of ARFID.

## Supplementary material

10.1136/bmjopen-2024-095559 online supplemental file 1
