# Supplementary material for: ARFID InitiativE Sweden (ARIES): study protocol for a large-scale genetic and registry-linked cohort study on avoidant/restrictive food intake disorder
Source: BMJ Open. 2025 Apr 17;15(4):e095559. doi: 10.1136/bmjopen-2024-095559 (PMC12007039; doi:10.1136/bmjopen-2024-095559)
Supplement: online supplemental file 1 [file bmjopen-15-4-s001.pdf]

Table S1. ARIES Assessment Battery

Domain	Assessment	Version	Parent- or self-report	Age	Mandatory vs. optional
**ARFID symptoms and diagnosis**					
ARFID symptoms	Nine Item ARFID Screen (NIAS) [1]	Parent version	Parent	6-14	Mandatory
			Self	12-14	Optional
ARFID symptoms	Pica, ARFID, and Rumination Disorder Interview-ARFID-Questionnaire (PARDI-AR-Q) [2]	Parent version + additional own questions	Parent	6-14	Mandatory
Drivers of food avoidance	Pica, ARFID, and Rumination Disorder Interview (PARDI) [3]	Selected and adapted items	Parent	6-14	Mandatory
		Selected and adapted items	Self	12-14	Optional
	Food Neophobia Scale (FNS) [4]	Selected items	Parent	6-14	Mandatory
		Selected items	Self	12-14	Optional
**Other ED pathology**					
Current ED symptoms (last 28 days)	Eating Disorder Examination-Questionnaire (EDE-Qv6) [5]	Parent Version 2.0 (PEDE-Qv2.0) [6]	Parent	6-14	Mandatory
		Brief version of the EDE-Q (EDE-Q-13) adjusted to teenagers [7]	Self	12-14	Optional
	Loss of Control over Eating Scale- Brief Version (LOCES-B) [8]	Selected and adapted items	Parent	6-14	Mandatory
Body dissatisfaction	Figure Rating Scale (FRS) [9]		Self	12-14	Optional

Food Intake	Swedish Food Frequency Questionnaire/ MiniMeal-Q* [10 11]		Parent	6-14	Mandatory
Pica & rumination disorder	PARDI [3]	Selected and adapted items	Parent	6-14	Mandatory
**Impairment**					
Mealtime interactions	Behavioral Pediatric Feeding Assessment Scale (BPFAS) [12]	Parent subscale, selected items	Parent	6-14	Mandatory
Impact on family	What Matters to Me (WMtM) [13]	Selected items	Parent	6-14	Mandatory
	PARDI [3]	Selected items	Parent	6-14	Mandatory
Treatment history	Own questions		Parent	6-14	Mandatory
Quality of life/severity	Own questions		Parent	6-14	Mandatory
			Self	12-14	Optional
**Co-occurring conditions**					
Anxiety, depression, and OCD	Revised Child Anxiety and Depression Scale (RCADS) [14]	RCADS-25 - caregiver version* [15 16]	Parent	6-14	Mandatory
		RCADS-25* [16 17]	Self	12-14	Optional
Autism	Autism-Tics, AD/HD and other comorbidities inventory* (A-TAC) [18]	Autism subscale	Parent	6-14	Mandatory
Attention deficit hyperactivity disorder (ADHD)	Swanson, Nolan, and Pelham Rating Scale (SNAP-IV) [19]		Parent	6-14	Mandatory
Other neurodevelopmental conditions	A-TAC* [18]	Selected and adapted items	Parent	6-14	Mandatory
	Own questions		Parent	6-14	Mandatory

Health conditions and medication	Own questions		Parent	6-14	Optional
Gastrointestinal symptoms	Own questions		Parent	6-14	Optional
			Self	12-14	Optional
**Environmental exposures**					
Life events	Linköping Youth Life Experience Scale (LYLES)* [20]	Adapted version	Parent	6-14	Optional
Pregnancy and birth	Selected questions from the ARFID-GEN pregnancy questionnaire		Parent	6-14	Optional
**Other**					
Temperament	One-item temperament scale [21]		Parent	6-14	Optional
Parental stress and EDs	Perceived Stress Scale (PSS-4)* [22 23]		Parent	6-14	Optional
	NIAS [1]		Parent	6-14	Optional
	PARDI-AR-Q [2]	Selected items	Parent	6-14	Optional
	ED100k [24]	Selected and adapted items	Parent	6-14	Optional
	7-Item Binge-Eating Disorder Screener (BEDS-7) [25]	Selected items	Parent	6-14	Optional
Priority setting	Own questions		Parent	6-14	Mandatory

* Validated Swedish version available

REFERENCES

1. Zickgraf HF, Ellis JM. Initial validation of the Nine Item Avoidant/Restrictive Food Intake disorder screen (NIAS): A measure of three restrictive eating patterns. *Appetite* 2018;123:32-42.
2. Bryant-Waugh R, Stern CM, Dreier MJ, et al. Preliminary validation of the pica, ARFID and rumination disorder interview ARFID questionnaire (PARDI-AR-Q). *Journal of Eating Disorders* 2022;10 (1):179.
3. Bryant-Waugh R, Micali N, Cooke L, et al. Development of the Pica, ARFID, and Rumination Disorder Interview, a multi-informant, semi-structured interview of feeding disorders across the lifespan: A pilot study for ages 10-22. *Int J Eat Disord* 2019;52 (4):378-87.
4. Pliner P, Hobden K. Development of a scale to measure the trait of food neophobia in humans. *Appetite* 1992;19 (2):105-20.
5. Fairburn CG, Beglin S. Eating disorder examination questionnaire (EDE-Q 6.0). In: Fairburn CG, ed. Cognitive behavior therapy and eating disorders. New York, NY: Guilford Press 2008:309–13.
6. Drury CR, Hail L, Rienecke RD, et al. Psychometric properties of the Parent Eating Disorder Examination Questionnaire. *Int J Eat Disord* 2023
7. Lev-Ari L, Bachner-Melman R, Zohar AH. Eating Disorder Examination Questionnaire (EDE-Q-13): expanding on the short form. *J Eat Disord* 2021;9 (1):57.
8. Latner JD, Mond JM, Kelly MC, et al. The Loss of Control Over Eating Scale: development and psychometric evaluation. *Int J Eat Disord* 2014;47 (6):647-59.
9. Collins ME. Body figure perceptions and preferences among preadolescent children. *Int J Eat Disord* 1991;10:199-208.
10. Christensen SE, Möller E, Bonn SE, et al. Two new meal- and web-based interactive food frequency questionnaires: validation of energy and macronutrient intake. *J Med Internet Res* 2013;15 (6):e109.
11. Delisle Nystrom C, Henriksson H, Alexandrou C, et al. Validation of an Online Food Frequency Questionnaire against Doubly Labelled Water and 24 h Dietary Recalls in Pre-School Children. *Nutrients* 2017;9 (1)
12. Crist W, Napier-Phillips A. Mealtime behaviors of young children: a comparison of normative and clinical data. *J Dev Behav Pediatr* 2001;22 (5):279-86.
13. Bryant-Waugh R. ARFID Avoidant Restrictive Food Intake Disorder. A guide for parents and carers. Abingdon, Oxon: Routledge 2020.
14. Chorpita BF, Yim L, Moffitt C, et al. Assessment of symptoms of DSM-IV anxiety and depression in children: a revised child anxiety and depression scale. *Behav Res Ther* 2000;38 (8):835-55.
15. Ebesutani C, Korathu-Larson P, Nakamura BJ, et al. The Revised Child Anxiety and Depression Scale 25–Parent Version: Scale Development and Validation in a School-Based and Clinical Sample. *Assessment* 2017;24 (6):712-28.
16. Carlander A, Cassel S, J-Son Höök M, et al. Validation and normative data on the Revised Child Anxiety and Depression Scale RCADS-25 in a Swedish national probability sample of children and adolescents aged 4–17 years. *Int J Methods Psychiatr Res* 2024;33 (1):e2007.
17. Ebesutani C, Reise SP, Chorpita BF, et al. The Revised Child Anxiety and Depression Scale-Short Version: scale reduction via exploratory bifactor modeling of the broad anxiety factor. *Psychol Assess* 2012;24 (4):833-45.
18. Larson T, Anckarsater H, Gillberg C, et al. The autism--tics, AD/HD and other comorbidities inventory (A-TAC): further validation of a telephone interview for epidemiological research. *BMC Psychiatry* 2010;10 (1):1.

19. Swanson JM, Kraemer HC, Hinshaw SP, et al. Clinical Relevance of the Primary Findings of the MTA: Success Rates Based on Severity of ADHD and ODD Symptoms at the End of Treatment. *J Am Acad Child Adolesc Psychiatry* 2001;40 (2):168-79.
20. Nilsson D, Gustafsson PE, Larsson JL, et al. Evaluation of the linköping youth life experience scale. *J Nerv Ment Dis* 2010;198 (10):768-74.
21. Sleddens EF, Hughes SO, O'Connor TM, et al. The Children's Behavior Questionnaire very short scale: psychometric properties and development of a one-item temperament scale. *Psychol Rep* 2012;110 (1):197-217.
22. Cohen S. Perceived stress in a probability sample of the United States. 1988
23. Nordin M, Nordin S. Psychometric evaluation and normative data of the Swedish version of the 10-item perceived stress scale. *Scand J Psychol* 2013;54 (6):502-7.
24. Thornton LM, Munn-Chernoff MA, Baker JH, et al. The Anorexia Nervosa Genetics Initiative (ANGI): Overview and methods. *Contemp Clin Trials* 2018;74:61-69.
25. Herman BK, Deal LS, DiBenedetti DB, et al. Development of the 7-Item Binge-Eating Disorder Screener (BEDS-7). *Prim Care Companion CNS Disord* 2016;18 (2)
