# Supplementary material for: ARFID InitiativE Sweden (ARIES): study protocol for a large-scale genetic and registry-linked cohort study on avoidant/restrictive food intake disorder
Source: BMJ Open. 2025 Apr 17;15(4):e095559. doi: 10.1136/bmjopen-2024-095559 (PMC12007039; doi:10.1136/bmjopen-2024-095559)
Supplement: Supplementary file 2 [file bmjopen-2024-095559.reviewer_comments.pdf]

PEER REVIEW HISTORY

BMJ Open publishes all reviews undertaken for accepted manuscripts. Reviewers are asked to complete a checklist review form and are provided with free text boxes to elaborate on their assessment. These free text comments are reproduced below.

ARTICLE DETAILS

Title (Provisional)

ARFID InitiativE Sweden (ARIES): Study protocol for a large-scale genetic and registry-linked cohort study on avoidant/restrictive food intake disorder

Authors

Hog, Liv; Fundin, Bengt T.; Everett Palm, Erik; Billger, Annelie; Bulik, Cynthia M.; Abbaspour, Afrouz; Dinkler, Lisa

---

VERSION 1 - REVIEW

**Reviewer**	**1**
**Name**	**Bould, Helen**
**Affiliation**	**University of Bristol**
**Date**	**10-Feb-2025**
**COI**	**None**

This paper describes an important and timely study, currently underway, of 1500 6-14 year olds with ARFID and a comparator group of 500 age-matched controls, using questionnaire measures of eating disorders, record-linkage to enable follow up, genetic analysis and analysis of gut microbiome in the two groups. This work will provide a significant advance in our understanding of this relatively new diagnosis and may help identify potential interventions.

As this is a protocol of a study which is already underway, it is not relevant to request changes to the methodology. I do not think there are major flaws in the study which would prevent sound interpretation of the data. I note that the numbers involved will be relatively small for the GWAS aspects of the study (though in combination with the previous GWAS with which results will be combined it will nonetheless be the largest GWAS of ARFID to date) and results from that part will have to be interpreted with caution.

My only note on the current manuscript is the usefulness of the current hypothesis in relation to Aim 2 "Conduct a within-disorder ARFID GWAS" - which is given as "We will identify genome-wide significant loci for ARFID". I think the authors should either acknowledge that this aim is exploratory/descriptive rather than hypothesis-driven, or alternatively hypothesise where they might expect to see genome-wide significant loci.

**Reviewer**	**2**
**Name**	**Sanchez-Cerezo, Javier**
**Affiliation**	**Imperial College London, Division of Psychiatry**
**Date**	**17-Feb-2025**
**COI**	**None**

Thank you very much for the opportunity to review this study protocol entitled 'ARFID InitiativE Sweden (ARIES): Study protocol for large-scale genetic studies of ARFID'. I commend the authors for submitting this study protocol for publication.

The ARIES study aims to identify genetic, environmental and microbial factors associated with ARFID. The study design is adequate to test the hypothesis. The planned statistical analyses are correct and appropriate.

The manuscript is well-written and appropriately structured.

For the editor:

• Protocol papers should report planned or ongoing studies. The dates of the study should be included in the manuscript.

o Study recruitment began in May 2024, and expected closing recruitment in summer 2025.

• While some baseline data can be presented, there should be no results or conclusions present in the study protocol.

o No results/conclusions included in the study protocol.

Minor comments:

Page 10: Methods section, Parent Advisory Council subsection: correct PCA.

Page 11: Methods section, Procedure subsection: are the questionnaires validated in Swedish or will they be completed in English?

Page 15: Methods section, Additional data sources subsection: for people working out of Sweden, how will the participants be linked to the registers? Will names or other identifiers be included in the data?

Page 17 Methods section, Phenotypic analyses subsection: remove demographics (duplicated).

VERSION 1 - AUTHOR RESPONSE

Reviewer 1

COMMENTS TO AUTHORS
This paper describes an important and timely study, currently underway, of 1500 6-14 year olds with ARFID and a comparator group of 500 age-matched controls, using questionnaire measures of eating disorders, record-linkage to enable follow up, genetic analysis and analysis of gut microbiome in the two groups. This work will provide a significant advance in our understanding of this relatively new diagnosis and may help identify potential interventions.

We would like to thank the reviewer for this comment.

As this is a protocol of a study which is already underway, it is not relevant to request changes to the methodology. I do not think there are major flaws in the study which would prevent sound interpretation of the data. I note that the numbers involved will be relatively small for the GWAS aspects of the study (though in combination with the previous GWAS with which results will be combined it will nonetheless be the largest GWAS of ARFID to date) and results from that part will have to be interpreted with caution.

We agree that the sample size for the GWAS component is relatively small in comparison to other disorders for which genetic data collections were started years or even decades ago. By combining ARIES data with other completed and ongoing data collections, we will maximize statistical power for the GWAS meta-analysis and ensure the most robust analysis possible. We added the following sentence to the limitation section:

Limitations: "Although the GWAS sample size will be relatively small, integrating ARIES data with other ongoing collections globally will maximize statistical power for a coordinated GWAS meta-analysis, ensuring the most comprehensive and robust analysis to date and resulting in the largest ARFID GWAS conducted so far."

My only note on the current manuscript is the usefulness of the current hypothesis in relation to Aim 2 "Conduct a within-disorder ARFID GWAS" - which is given as "We will identify genome-wide significant loci for ARFID". I think the authors should either acknowledge that this aim is exploratory/descriptive rather than hypothesis-driven, or alternatively hypothesise where they might expect to see genome-wide significant loci.

We thank the reviewer for pointing this out. We agree and have clarified that GWAS is a hypothesis-free method while maintaining our expectation of identifying genome-wide significant loci for ARFID. The revised text is as follows:

Aim 2, hypothesis: "GWAS is a hypothesis-free method to identify genetic loci across the entire genome that are statistically associated with risk for a trait or a condition. We expect to identify genome-wide significant loci for ARFID."

Reviewer 2

COMMENTS TO AUTHORS
Thank you very much for the opportunity to review this study protocol entitled 'ARFID InitiativE Sweden (ARIES): Study protocol for large-scale genetic studies of ARFID'. I commend the authors for submitting this study protocol for publication.
The ARIES study aims to identify genetic, environmental and microbial factors associated with ARFID. The study design is adequate to test the hypothesis. The planned statistical analyses are

correct and appropriate.
The manuscript is well-written and appropriately structured.

We appreciate the reviewer's comment.

Page 10: Methods section, Parent Advisory Council subsection: correct PCA.

We have corrected the abbreviation error in the Methods section, Parent Advisory Council subsection, and have used the correct abbreviation.

Parent Advisory Council (PAC): "Prior to the main study launch, the PAC completed a beta version of the entire study, including all questionnaires and saliva sampling, and provided feedback on all study procedures."

Page 11: Methods section, Procedure subsection: are the questionnaires validated in Swedish or will they be completed in English?

Thank you for pointing out the need for clarification. All questionnaires used in the study are administered in Swedish. While some of the translations have been used in previous research, only a few have undergone formal validation in Swedish. We have added a clarification in the manuscript and indicated which questionnaires have validated Swedish versions in Table S1: ARIES Assessment Battery.

Assessment battery: "Questionnaires are applied in Swedish and have been adapted for administration via Forsta (previously Confirmit)."

Page 15: Methods section, Additional data sources subsection: for people working out of Sweden, how will the participants be linked to the registers? Will names or other identifiers be included in the data?

We thank the reviewer for the questions. Participants must have a Swedish personal number (a unique national identification number) to be included in the study, as only those with a valid Swedish personal number can provide consent on the study website (via BankID, a secure identity verification system) and be linked to the Swedish National Health Registers. The register linkage is conducted by Swedish government agencies. We do not include personally identifying information such as names in the study data, i.e., for data analyses, personal numbers will be replaced with a study ID. We added the following clarifications:

Procedure: "Parents/guardians of children and adolescents with ARFID who are interested in participation will be referred to the study website (www.ariesstudy.se) that provides detailed study information and contact details in case of questions ("parents/guardians" will henceforth be referred to as "parents" for readability). To be eligible for participation, all individuals must have a valid Swedish personal number (a unique national identification number), which is required for both providing consent and linking to the Swedish National Health Registers."

Additional data sources: "Our data will be linked to nationwide Swedish health registers including the National Patient Register (NPR) [43], the Prescribed Drug Register (PDR) [45], Medical Birth Register (MBR) [44], and the National Quality Register for Specialized Eating Disorder Treatment (Riksät/Stepwise) [46]. Register linkage is conducted by government agencies via the Swedish personal number."

Data analysis: “Research data will be pseudonymized for data analyses. We will follow pre-determined statistical analysis plans for phenotype, genotype, and gut microbiome data. All analyses will include age and sex/gender as covariates and be corrected for multiple testing.”

Page 17 Methods section, Phenotypic analyses subsection: remove demographics (duplicated).

We removed the duplicate:

“We will describe the ARFID sample to present ~~demographics,~~ demographics; ARFID symptoms, onset, and course; other ED pathology; food intake; co-occurring psychiatric, neurodevelopmental, and somatic conditions and traits; treatment utilization; environmental exposures; impairment and quality of life; and parental stress and ED pathology.”
